# Subtle Infantile Hemiparesis as a Presentation of Acute Disseminated Encephalomyelitis: The Value of Caregiver Video Documentation

**DOI:** 10.1002/ccr3.70900

**Published:** 2025-09-21

**Authors:** Hideki Tanoue, Sayaka Ishihara, Mari Asakura, Masahiro Noda, Kunihiro Oba, Masashi Ogasawara

**Affiliations:** ^1^ Department of Pediatrics Showa General Hospital Kodaira Japan; ^2^ Department of Neuromuscular Research, National Institute of Neuroscience National Center of Neurology and Psychiatry (NCNP) Kodaira Japan

**Keywords:** acute disseminated encephalomyelitis, child, encephalopathy, mild hemiparesis, MOG antibody‐associated disease

## Abstract

Subtle neurological symptoms, such as mild hemiparesis, may represent the earliest signs of acute disseminated encephalomyelitis (ADEM) in infants. This case illustrates how caregiver‐recorded videos can provide crucial diagnostic clues by capturing functional changes that might otherwise remain unnoticed during routine clinical observations.

1

A 1‐year‐old boy presented with fever and subtle right‐hand weakness. Although the patient remained alert (GCS E4V5M6) and ambulatory, his caregiver reported decreased use of the right hand, lethargy, and prolonged sleep (Figure 1A). On initial examination, he was able to grasp objects spontaneously and wave bye‐bye with both hands. At a follow‐up visit 2 days later, he was still alert and able to walk. The caregiver‐recorded video documented that his coordination was age‐appropriate. However, right‐hand movements were slower and less efficient (Video 1).

**FIGURE 1:**
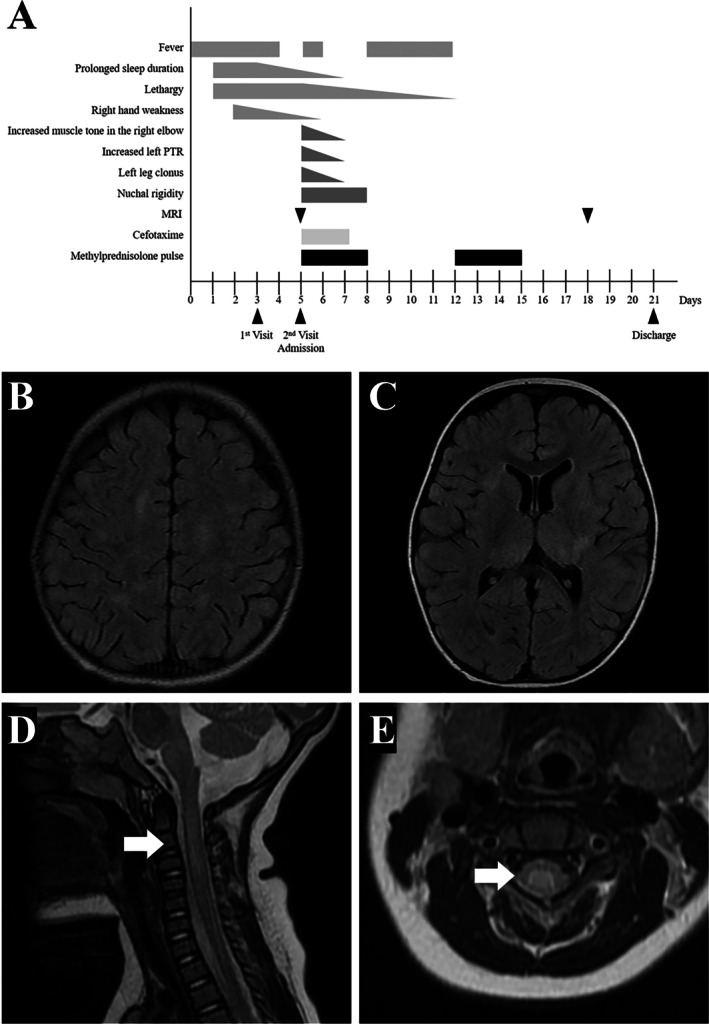
(A) Clinical course of the patient. Horizontal bars indicate the duration of symptoms and treatments. Fever, limb weakness, prolonged sleep duration, and lethargy were observed during the first week. At the second visit, he was still alert and able to walk but exhibited slightly increased muscle tone in the right elbow, left leg clonus, and hyperreflexia of the left patellar tendon. Methylprednisolone pulse therapy was administered twice (Days 5–7 and 12–14). MRI was performed on Days 5 and 18. The patient was discharged on Day 21. (B, C) Brain FLAIR images showing high‐intensity signals in the subcortical and deep white matter. (D, E) Cervical spinal cord MRI (T2‐weighted images) showing high signal intensity in the central gray matter at the C2–C6 levels (arrows).

**VIDEO 1:** (Affected) While the patient was affected, he was able to eat with a spoon; however, he had difficulty using his right hand and exhibited right‐hand bradykinesia. At 00:10, the patient exhibited compensatory elbow elevation due to weakness of the wrist extensors. At 00:13, the patient leaned forward to assist with self‐feeding. (Before onset) The patient could eat food quickly and smoothly and did not lean forward. At 00:25, the patient was able to eat while maintaining an upright position. Brain MRI revealed high‐intensity signals in the subcortical and deep white matter on FLAIR images. Video content can be viewed at https://onlinelibrary.wiley.com/doi/10.1002/ccr3.70900.

Brain and cervical MRI on Day 5 revealed multifocal T2WI/FLAIR hyperintense lesions (Figure 1B–E and Video 1). Serum anti‐MOG antibody was positive. Based on these findings, the patient was diagnosed with ADEM. He was treated with intravenous methylprednisolone and achieved complete neurological recovery within 2 weeks.

Diagnosing ADEM in infants is challenging because the symptoms may be subtle and limited in expression [1]. This case underscores the clinical importance of caregiver‐recorded videos in identifying subtle neurological signs in infants.

## Author Contributions

**Hideki Tanoue:** data curation, investigation, resources, writing – original draft, writing – review and editing. **Sayaka Ishihara:** data curation, investigation, resources, writing – review and editing. **Mari Asakura:** supervision, writing – review and editing. **Masahiro Noda:** supervision, writing – review and editing. **Kunihiro Oba:** data curation, investigation, resources, supervision, writing – review and editing. **Masashi Ogasawara:** data curation, investigation, resources, supervision, writing – original draft, writing – review and editing.

## Conflicts of Interest

The authors declare no conflicts of interest.

## Data Availability

The data supporting the findings in this study is available from the corresponding author upon request.
